# The Rise of the Data Poor: The COVID-19 Pandemic Seen From the Margins

**DOI:** 10.1177/2056305120948233

**Published:** 2020-08-11

**Authors:** Stefania Milan, Emiliano Treré

**Affiliations:** 1 University of Amsterdam, The Netherlands; 2 Cardiff University, UK

**Keywords:** data poverty, COVID-19 pandemic, data gaps, critical data studies, margins

## Abstract

Quantification is central to the narration of the COVID-19 pandemic. Numbers determine the existence of the problem and affect our ability to care and contribute to relief efforts. Yet many communities at the margins, including many areas of the Global South, are virtually absent from this number-based narration of the pandemic. This essay builds on critical data studies to warn against the universalization of problems, narratives, and responses to the virus. To this end, it explores two types of data gaps and the corresponding “data poor.” The first gap concerns the data poverty perduring in low-income countries and jeopardizing their ability to adequately respond to the pandemic. The second affects vulnerable populations within a variety of geopolitical and socio-political contexts, whereby data poverty constitutes a dangerous form of invisibility which perpetuates various forms of inequality. But, even during the pandemic, the disempowered manage to create innovative forms of solidarity from below that partially mitigate the negative effects of their invisibility.

Since the COVID-19 virus was first identified in mainland China at the end of 2019, the pandemic has affected an exceptionally high portion of the world population. Not surprisingly, numbers are at the very core of the narration of the pandemic. Figures of various kinds fill the news, accounting for the death toll, the progress of population testing, the growth of individuals who tested positive for the virus, the saturation of intensive care units, among others. These numbers contribute to making the problem “amenable to thought” and thus serve as “both representation and intervention” (Osborne & Rose, 2004, p. 212). As such, they shape both governmental action and the popular response to it. Although they are not neutral or absolute, they are attributed a sort of “mechanical objectivity” (Porter, 1995) that positions the exercise of enumerating and comparing above other forms of knowing and feeling (see also Bowker & Star, 2000). In a nutshell, numbers determine the existence of the problem, and they determine which countries and social groups ought to elicit our concern. They affect our ability to care, to empathize, and to abide by the oftentimes draconian measures adopted in the effort to curb the pandemic. Yet many communities at the margins, including many areas of the so-called Global South, are virtually absent from this number-based narration of the pandemic.

Communities that remain in the shadows include but are not limited to undocumented migrants, refugees and people on the move, members of labor forces operating in submerged markets, and/or under precarious conditions such as sex workers, gig workers and farmhands, impoverished families, victims of domestic violence, but also developing countries with a suboptimal statistical and testing capacity. This has two main implications. First, the pandemic might exacerbate existing inequalities, aggravating the difficulties of populations at risks who are made even more invisible by mediocre monitoring and by exclusion from health care or welfare subsidies. Second, in the absence of reliable data, institutions, including governments in the South, might be tempted to “import” predictions and models from other socio-economic realities and base domestic measures on these, further ignoring invisible sectors of the population. The urge to “universalize” both problem and solutions—basing local policy on policy responses meant to address different contexts—overlooks the fact that numbers are deeply ingrained in specific socio-economic and political geographies.

In this essay, we build on critical data studies (cf. Dalton et al., 2016) to warn against the universalization of problems, narratives and responses (S. Milan & Treré, 2019), and encourage scholars to reflect on challenges of COVID-19, specifically when observed from the margins. “[T]he margin,” argues Rodríguez (2017), is “a shortcut to speak of complex dynamics of power inequality. Processes of asymmetrical access to material and symbolic resources shape differentiated and unequal access to the public sphere” (p. 49). We argue that this asymmetrical access is particularly virulent in the datafied society, which grounds the so-called public sphere in data generation, trade, and processing.

More specifically, the essay explores the widening data gaps of this pandemic, which largely maps into known, historical gaps in the economic and digital realms, and exposes how even during the pandemic, the disempowered manage to create innovative forms of solidarity from below that partially mitigate the negative effects of their invisibility.

## Two Types of Data Gaps

If numbers are at the core of the the COVID-19 problem, we ought to pay attention to who is represented in these numbers and who is (deliberately or not) left out. These “data gaps” concern both data generation and data quality, which even in ordinary times can jeopardize “evidence-based policy making, tracking progress and development, and increasing government accountability” (Chen et al., 2013, p. 1). Data gaps are a known weakness of the datafied society. Among others, boyd and Crawford (2012) warned against the “big data divide” in matter of ownership and access, while Manovich (2011) exposed the “data analysis divide” highlighting disparities in data usage and related skills. Reinterpreting the somewhat forgotten literature on the digital divide, which at the turn of the millennium provided a word of caution concerning the optimistic narratives associated with the digital revolution (e.g., Norris, 2001), McCarthy (2016) explains how this divide perpetuates severe “digital inequalities,” which affect a number of areas of human activity, including identity, self-determination, visibility, and agency.

The data gaps exacerbated by the pandemic, however, assume also another dramatic connotation. Rather than solely revealing “the asymmetric relationship between those who collect, store, and mine large quantities of data, and those whom data collection targets” (Andrejevic, 2014, p. 1673), these data gaps expose a new type of “data poverty” (to paraphrase boyd and Crawford)—one that is essentially a *sine qua non* condition of existence. It is no longer solely a matter of data exploitation (Zuboff, 2019) or data colonialism (Couldry & Mejias, 2018), but rather it gets to the bottom of what it means to be human. That is, data is tied to peoples’ visibility, survival, and care. Today’s “data poor” are not in opposition to the “Big Data rich” evoked by boyd and Crawford (2012). Rather, their concerns have to do with very fundamental types of inequality that pre-date the emergence of the datafied society but are possibly worsened by the policymakers’ over-reliance on “calculative publics” (Gillespie, 2014, p. 188), brought into existence by omnipresent data infrastructures.

We can identify at least two problematic situations related to this data poverty. The first concerns developing countries, while the second has to do with invisible populations within a variety of geopolitical and socio-political contexts.

### Data Poverty in Low-Income Countries

Facing an outbreak that knows no borders, the problems of developing countries vis-à-vis the pandemic are manifold (see, for example, Masiero, 2020). One of the worst case scenarios on the large scale relates to the (in)ability of many countries in the South, on the one hand, to produce reliable population statistics, and on the other, to test their population for the virus, due to the scarce availability of testing kits as well as adequate medical facilities (Diallo, 2020). The consequences of this data poverty are particularly harsh when lack of monitoring capabilities meets the absence of a nation-wide health system, like in the sub-Saharan region (Quaglio et al., 2020). To be sure, progress in population monitoring followed the revision of the United Nations’ Millennium Development Goals in 2005, when countries in the Global South have received support to devise National Strategies for the Development of Statistics (Chen et al., 2013). The urgency of the pandemic seems to have the positive effect of accelerating the response: the Regional Office for Africa of the World Health Organization (2020) reports that as of mid-May 2020, 44 countries in the region can test for COVID-19. There were only two countries on this list at the start of the outbreak.

Lack of reliable numbers to accurately portray the COVID-19 pandemic as it spreads to the Southern hemisphere might result in the dangerous equation “no data = no problem”—with consequences that transcend epidemiological considerations to affect society at its core. Most notably, it offers fertile ground for the spread of misinformation (or what has been termed an “infodemic,” see United Nations Department of Global Communications, 2020) as well as distorted narratives mobilized at the service of populist agendas. For example, Mexican left-wing populist president Andrés Manuel López Obrador responded to the coronavirus emergency insisting that Mexicans should “keep living life as usual,” and went as far as declaring that the pandemic is a plot to derail his presidency (Agren, 2020). On the opposite side of the political spectrum, Brazil’s far-right president Jair Bolsonaro dismissed the pandemic as a collective “hysteria,” notwithstanding the rising death toll (Phillips, 2020). The “fake news” that individuals of African origin are “immune” to the disease swept social media, in both Western countries and the African continent itself (Maclean, 2020). In Italy, the fact that most hospitalized patients are White while undocumented migrants have no access to health care has unleashed a plethora of racist comments and anti-migrant calls for action (Huffington Post, 2020)—which leads us to discuss a second form of data poverty.

### Data Poverty as a Form of Invisibility

A distinct instance of data poverty concerns many of the populations at the margins identified above, most notably undocumented foreign nationals, workers of informal economies and vulnerable populations in general, including the those who are homeless and gig workers. These segments of society suffer invisibility in ordinary times as well. Oftentimes, this invisibility is a blessing for those living at the margins who might, for example, put food on the table by engaging in informal or illegal activities. This is the case for some sex workers, who are often part of groups who are already marginalized, like people of color, or lesbian, gay, bisexual, and transgender (LGBT) individuals, for whom sex work might represent “one option among bad ones” (Wheeler, 2020).

During the pandemic, this invisibility translates into the virtual absence of “official” data about these groups—with two main consequences. On one hand, it means augmented risks for these people but also for their surrounding communities (S. Milan et al., 2020). On the other hand, it results in the absence of specific support measures also within resource-rich countries. For example, sex workers are typically excluded from pandemic-triggered recovery plans; operators of the shadow economy unable to work, often are not part of the count for unemployment subsidies. Furthermore, a mix of fear, social stigma, criminalization, and shortsighted legislation prevents individuals and social groups at the margins from coming forward when in need of care. In many such cases, invisibility might equal death, for example, in the case of victims of domestic violence (Villaseñor, 2020), or might trap people in the conditions that make them vulnerable in the first place.

## Countering Data Poverty: Collective Solidarities From Below

While institutional responses to these forms of invisibility have been varied and largely absent, the COVID-19 pandemic has exposed how vulnerable and marginalized groups have nonetheless managed to construct innovative forms of solidarity from below, which serve to soften the negative impact of their invisibility.

This is part of a counter-hegemonic emotional culture (Gravante & Poma, 2020) of collective solidarity, care and grassroots activism that signals “the desperate yearning of the population for some sense of solidarity amid the crisis” (Gerbaudo, 2020). Trying to overcome the absence or slowness of state action, grassroots groups have mobilized to support neighbors, elderly people, individuals with disability and long-term health conditions, precarious workers, indigenous communities, and counting. Mobilizations and activist groups have spurred in the Global South and high-income countries alike, ramping up the creation of mutual aid groups, strike actions, and solidarity networks to make visible the data poor and improve the conditions of marginalized groups during the pandemic. For instance, women’s collectives have expanded their reach within the community by distributing food, medicine, and essential products across Mexico (Ventura Alfaro, 2020) and China (Bao, 2020). Kenya is witnessing a resurgence of social movement activities, which provide alternative narratives of the crisis (Chukunzira, 2020). In China, activists have sought to bypass governmental censorship about the pandemic by documenting the spread of the virus on the software repository GitHub (“In Memory of COVID-19 in China: Various Forms of Digital Resistance Towards Censorship,” 2020).

New repertoires of action emerge to counter the effects of the lockdown imposed in many countries, which prevent people from taking the streets, with several actions going digital. Along the so-called Balkan route, solidarity with people on the move resulted in a 48-hr campaign called “A soap for IOM (International Organization for Migration),” denouncing the mismanagement of the refugee centers run by IOM in the region, which deprives migrants of basic rights (C. Milan, 2020). Chenoweth and colleagues (2020) have documented over 140 strategies of dissent and collective action specifically related to COVID-19. Their preliminary mapping displays the incredible richness of these novel online, offline and hybrid repertoires of contention, that include grassroots tactics of “data making” (Pybus et al., 2015) at the margins, where vulnerable groups and their allies become active producers and consumers of alternative narratives to reclaim their visibility amid the pandemic. Together, these forms of solidarity, protest, and resistance warn us against turning a blind eye to the impending forms of data poverty.
